# Predictors of anticipated coping behavior at myocardial infarction symptom onset among a nationwide sample of Korean adults

**DOI:** 10.4178/epih.e2021006

**Published:** 2021-01-03

**Authors:** Kyong Sil Park

**Affiliations:** School of Nursing, Cheju Halla University, Jeju, Korea

**Keywords:** Myocardial infarction, Heart attack, Cardiovascular disease, Emergency medical service, Behavior, Health Belief Model

## Abstract

**OBJECTIVES:**

This cross-sectional study based on the health belief model investigated predictors of anticipated coping behavior at myocardial infarction (MI) symptom onset using secondary data from the 2017 Korea Community Health Survey.

**METHODS:**

Modifying variables (socioeconomic, health knowledge, perceived threat) were selected as independent variables and anticipated coping behavior at MI symptom onset as the dependent variable. Calling 911 was classified as the correct anticipated coping behavior, while visiting a hospital or an oriental hospital, calling family, and others were classified as incorrect.

**RESULTS:**

Of 227,740 participants, 83.2% reported correct anticipated coping behaviors. The likelihood of calling 911 was low if participants experienced atypical symptoms (jaw, neck, back, arm, and shoulder pain), even if they were aware of those symptoms. However, 69.9% of participants who were aware of typical symptoms (chest pain) stated that they would call-911. Sex, age, hypertension, dyslipidemia, obesity, and awareness of MI symptoms affected the correct anticipated coping behavior.

**CONCLUSIONS:**

Correct coping abilities among the general public are vitally important for early treatment of MI patients and reduction of hospitalization time. Members of the general public in their 20s and 30s, 60 years of age or older, with cardiovascular risk factors (male sex, hypertension, dyslipidemia, and obesity), and who are not aware of MI symptoms should be educated about the typical and atypical symptoms of MI. Emergency medical services should be called without delay if needed, and public relations activities should be carried out to raise awareness that anyone can use emergency medical services.

## INTRODUCTION

The mortality rate of cardiovascular disease (CVD) in Korea is increasing, with approximately 62 deaths per 100,000 population in 2018. In particular, the mortality from ischemic heart disease including myocardial infarction (MI) and angina pectoris accounts for approximately 50% of deaths from heart disease [1]. Because MI can cause diverse complications and disorders as well as death, prompt coping at the onset of MI symptoms is the most crucial factor in determining patient prognosis. The use of an emergency medical service (hereafter, 911) has been identified as an independent influential factor in lowering the mortality due to MI [3]. Thus, when a patient shows MI symptoms, an immediate call to 911 and the appropriate use of the 911 service is believed to be critical in reducing the likelihood of death and improving the prognosis.

It was reported, however, that approximately 44% of patients arrive at the hospital within 2 hours after the onset of MI symptoms [4] and approximately 47.7% within 3 hours, indicating that more than 50% of patients arrive at the hospital outside the golden hour [5]. Inappropriate patient transportation, such as the use of a personal car or contacting the family, causes a delay in the hospital arrival [4], which leads to the loss of the crucial opportunity to treat MI (i.e., the golden hour), affecting the patient’s likelihood of survival [6]. Studies have shown that only 28.8% of MI patients used the 911 service, that they used the service only when in severe pain [7], and that they had incorrect perceptions of the service and felt embarrassed or uncomfortable using it [8].

It is important not only for patients but also for families and the general public to have the correct knowledge on coping to MI symptoms so that the patients can receive the appropriate treatment within the golden hour [9]. A recent study conducted with the general public reported that the study participants perceived chest pain as the most typical among the five representative MI symptoms but showed poor awareness of radiating pain (i.e., pain spreading to the shoulders and arms). Additionally, for anticipated coping behavior, approximately 83% of the participants made the correct choice (calling 911) [10]. In comparison to a 2019 finding of a response rate of 95.5% in the United States [11], the finding above shows that the knowledge of the coping behavior at the onset of MI symptoms is still low in Korea.

In contrast, public institutions have actively provided cardiopulmonary resuscitation (CPR) training to the general public and also held campaigns targeting the entire population. As a consequence, the rate of CPR performance by bystanders markedly increased from 1.9% in 2008 to 23.5% in 2018 [12]. However, challenges remain in the bystanders’ CPR performance because the general public do not have specialized knowledge and are afraid of performing CPR [13]. In addition, it was reported that the longer ago they received CPR training, the less likely they were to perform CPR [14]. Therefore, it is necessary to examine various characteristics of the general public and provide CPR training and campaigns according to their characteristics.

In the Unite States, research has been actively conducted with the general public regarding appropriate coping behaviors at the onset of MI symptoms. The relationship between the awareness of early MI symptoms and appropriate coping behavior was examined using socio-demographic factors [11], and being married and hypertensive and having the knowledge of early MI symptoms were identified as predictors of appropriate coping behaviors in response to MI symptoms [15].

However, MI research in Korea has been conducted primarily focusing on MI patients. With MI patients as the study participants, some studies examined MI symptom recognition, hospital arrival rate, and factors influencing delays in hospital arrival [4,16], and some other studies demonstrated the effects of training programs for CVD and CRP on knowledge and likelihood to perform CPR [17]. Research has also been conducted with the general public to examine the level of awareness of MI symptoms [10] and the effects of CVD and CPR training programs on the likelihood to perform CPR [16]. But studies which identified factors influencing anticipated coping behavior in response to MI symptoms have been conducted only with MI patients, and no studies have been conducted with the general public.

It is crucial not only for patients with MI but also for the general public in the community to appropriately cope at the onset of MI symptoms so that patients may receive appropriate treatment within the golden hour [10]. Accordingly, it would be necessary to examine anticipated coping behaviors of the general public in the community and identify factors affecting the coping behavior so that when a patient shows MI symptoms, the first witness can immediately and appropriately cope to shorten the length of the pre-hospital phase. Thus, this study aimed to investigate the general public’s anticipated coping behavior in response to MI symptoms. An additional purpose of the study was to identify the factors related to the anticipated coping behavior, based on the Health Belief Model (HBM), a model developed to explain behavioral changes in humans [18], by matching the study participants’ characteristics to each of the components of the model.

## MATERIALS AND METHODS

### Research design

This study was a cross-sectional survey using the data from the 2017 Korea Community Health Survey (CHS) to examine the general public’s anticipated coping behavior at the onset of MI symptoms and identify factors influencing the anticipated coping behavior.

### Study participants

The study utilized the 2017 CHS raw data of 228,381 participants. Data on the items regarding MI symptoms and regarding anticipated coping behavior in response to MI symptoms were missing in 100 and 541 participants, respectively; these participants were excluded from the study. Thus, a total of 227,740 participants were included.

### Measurements

The HBM has been used as an important predictor in explaining health-related behavior, such as smoking, exercise, and health service utilization, and lately is used to predict behavior in diverse areas [18]. Based on a previous study which used the CHS data [14], the demographics, health knowledge, and perceived threat (i.e., modifying factors) were used as independent variables and anticipated coping behavior was chosen as dependent variable, as it was infeasible to examine all components of the HBM.

Socio-demographics included sex, age, education level, marital status, occupation, and income. Moreover, the awareness of early MI symptoms was selected for the structural factors. For perceived threats, hypertension, diabetes, dyslipidemia, and obesity were selected. Occupation was categorized into professional administrative managers and office workers (managers, professionals and related workers, and clerks), sales/service workers and craft/elementary workers (service workers; sales workers; skilled agricultural, forestry, and fishery workers; craft and related trades workers; equipment, machine operating, and assembling workers; elementary workers and armed forces), and the unemployed and homemakers (the unemployed, homemakers, and students). Of the major risk factor of CVD, such as hypertension, diabetes, and dyslipidemia, patients were classified as having the corresponding condition if the response to either “diagnosis by a physician” or “current treatment” was “yes.” On the basis of the CHS guidelines, hypertension was defined as a systolic blood pressure ≥ 140 mmHg or a diastolic blood pressure ≥ 90 mmHg. Diabetes was defined as a fasting blood glucose level ≥ 126 mg/dL, 2-hour glucose tolerance test ≥ 200 mg/dL, or glycated hemoglobin ≥ 6.5%. Furthermore, dyslipidemia was defined as low density lipoprotein ≥ 160 mg/dL, high density lipoprotein < 40 mg/dL, total cholesterol ≥ 240 mg/dL, or triglycerides ≥ 200 mg/dL [19]. Lastly, obesity was defined as body mass index (BMI) ≥ 25 kg/m^2^ [20,21].

Knowledge of the disease corresponds to the structural factor in the HBM [18]. In this study, the knowledge of early MI symptoms was selected for knowledge of the disease. The relevant question was “which of the following symptoms do you think is an MI symptom?” and the items were as follows: (1) pain or discomfort in the jaw, neck, or back; (2) weakness, light-headedness, nausea, or cold sweat; (3) pain, pressure, or squeezing in the chest; (4) sudden pain or discomfort in the arms or shoulders; and (5) sudden shortness of breath. Each of the items was responded with “yes” or “no.” Participants who responded with “do not know” to the items regarding MI symptoms were considered not having knowledge of MI and their responses were recoded to “no.” Those who checked “refuse to respond” were excluded from the study since they were treated as cases with missing data.

The dependent variable was anticipated coping behavior at the onset of MI symptoms was chosen. The relevant item was “what do you think is the first thing you should do when someone shows an MI symptom?” In accordance with the guidelines by the Korea Centers for Disease Control and Prevention (KCDC) [22], “call-911” was categorized as the appropriate coping behavior, while “go to hospital,” “go to oriental hospital,” “contact the family,” and “others” were classified as inappropriate.

### Statistical analysis

Data were analyzed using IBM SPSS version 20.0 (IBM Corp., Armonk, NY, USA). Below, frequencies and percentages are presented for nominal variables and means and standard deviations for continuous variables. Chi-square test was performed to examine anticipated coping behavior in response to MI symptoms, and multivariate logistic regression to identify factors influencing the anticipated coping behavior. Statistical significance was determined as p-value < 0.05.

### Ethics statement

A request for raw data was made through the CHS website. After approval, the CHS provided the researcher with the raw data without study participants’ personal information.

## RESULTS

Regarding socio-demographic characteristics of participants, there were more females (55.1%) than males, and the most common age group was 60 or older (39.1%). Moreover, 69.6% of the participants had high school education or less, and 67.5% were married. For occupation, 43.6% were sales/service or craft/elementary workers, and 36.0% had a monthly income less than 2 million Korean won (KRW). With respect to the major risk factor of CVD, 27.3% had hypertension, 11.0% diabetes, 17.4% dyslipidemia, and 26.3% a BMI over 25 kg/m^2^. Of the participants, 58.8% correctly recognized at least four out of the five major MI symptoms (Table 1).

For the anticipated coping behavior in response to MI symptoms, participants in the call-911 group were compared with those in all other groups. The result showed that the proportion of participants in the call-911 group was higher for those who were 40 years or older; had high school education or less; were married; were a sales/service worker or a craft/elementary worker; had a monthly income less than 2 million KRW; had hypertension, diabetes, dyslipidemia, or obesity; and correctly recognized early MI symptoms (Table 2).

**Figure 1.**
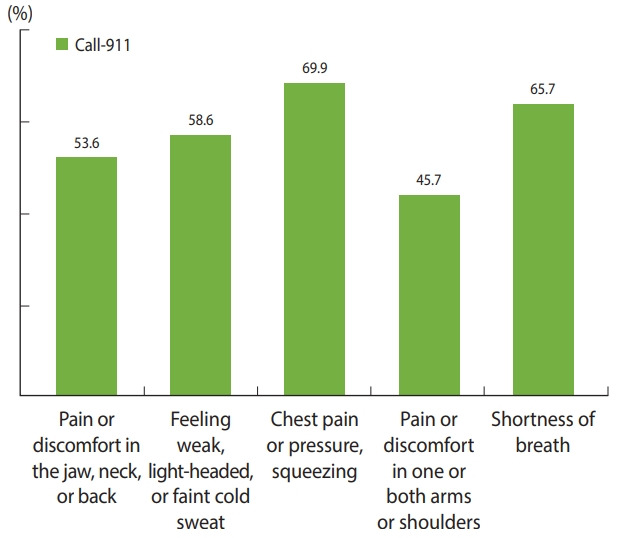
Comparison of correct anticipated coping behavior (call911) with each symptom of myocardial infarction.

Anticipated coping behavior was examined for each of the five major MI symptoms and the results showed that the proportion of participants who would call-911 was 53.6% of those who recognized “pain or discomfort in the jaw, neck, or back;” 58.6% of those who recognized “weakness, light-headedness, nausea, or cold sweat;” 69.9% of those who recognized “pain, pressure, or squeezing in the chest;” 45.7% of those who recognized “sudden pain or discomfort in the arms or shoulders;” and 65.7% of those who recognized “sudden shortness of breath” (Figure 1).

The analysis conducted to identify factors influencing anticipated coping behavior in response to MI symptoms showed that the anticipated coping behavior was higher in female compared to male (odds ratio [OR], 1.02; 95% confidence interval [CI], 1.00 to 1.05). Anticipated coping behavior was higher in the 40–59 age group (OR, 1.07; 95% CI, 1.04 to 1.11) and lower in the 60 or older age group (OR, 0.94; 95% CI, 0.90 to 0.97), relative to the 20-39 age group. Anticipated coping behavior was higher in participants with hypertension (OR, 1.06; 95% CI, 1.03 to 1.08), with dyslipidemia (OR, 1.05; 95% CI, 1.02 to 1.09), and with a BMI over 25 kg/m^2^ (OR, 1.04; 95% CI, 1.02 to 1.07) than in participants without the condition. Furthermore, anticipated coping behavior was significantly higher in the participants who recognized two or three of the five major MI symptoms (OR, 1.23; 95% CI, 1.19 to 1.27) as well as in the participants who recognized four or five of the symptoms (OR, 1.46; 95% CI, 1.41 to 1.50) than in participants who recognized only one or none of the symptoms (Table 3).

## DISCUSSION

In this study, 83.2% of the participants chose to call-911 as the anticipated coping behavior in response to MI symptoms, which is the appropriate coping behavior. This level was higher than the 2010 finding of 67% [23] and the finding may be interpreted as a consequence of the nationwide education and awareness campaigns. However, the level is lower than the rate of calling 911 in the United States, which is 95.5% [11]. Moreover, approximately 16.8% selected inappropriate coping behavior such as going to hospital or oriental hospital and contacting the family, which would delay the patient’s hospital arrival. It would be necessary to provide training and campaigns stressing that the 911 ambulance services are not mere transportation vehicles but a crucial primary medical service offering diagnosis and treatment [7].

In the analysis conducted to identify factors affecting the general public’s anticipated coping behavior in response to MI symptoms, the awareness of early MI symptoms was identified as the most influential predictor of the anticipated coping behavior. It has been reported that in Hong Kong, the awareness of early MI symptoms was found to influence inappropriate anticipated coping behavior [15], a finding consistent with the current study finding. According to the analysis of appropriate anticipated coping behavior by the awareness of each of the major MI symptoms, only 53.6% of those who were aware of the pain in the jaw, neck, or back and only 45.7% of those who were aware of the pain in the arms or shoulders chose calling 911 as the anticipated coping behavior. In contrast, the proportion of participants who chose calling 911 was the highest, 69.9%, in those who recognized chest pain as an MI symptom. Thus, it can be inferred that it is likely for bystanders to perform inappropriate coping behavior even if they are well aware of the MI symptoms, and the likelihood of performing inappropriate coping behavior is higher in response to atypical symptoms than typical ones. Likewise, a previous study found that the recognition of chest pain and neck pain was a predictor of inappropriate anticipated coping behavior [15]. It was reported in a qualitative study with MI patients that patients lacked an understanding of atypical symptoms and had incorrect perceptions regarding the use of emergency medical service, perceiving that using the service was embarrassing or uncomfortable [8]. Moreover, there was a misperception that 911 ambulance vehicles should be used only in the presence of severe pain [7]. Accordingly, to promote the appropriate coping behavior at the onset of MI symptoms (i.e., calling 911), atypical MI symptoms as well as typical symptoms should be emphasized in MI education and training, and campaigns should be aggressively held to raise the awareness among the general public that anyone can call-911 and utilize the service in an emergency.

With respect to the perceived risks, hypertension, dyslipidemia, and obesity were identified as predictors of the coping behavior in response to MI symptoms. A previous study reported that hypertension was a significant factor in influencing inappropriate treatment-seeking behaviors [15] and in another study, participants with MI or angina pectoris were more likely to perform CPR [14]. Although it is difficult to make a direct comparison, in yet another previous study, hypertension, dyslipidemia, stroke, MI, and angina pectoris were identified as influencing the level of awareness of CVD [24]. According to the HBM, perceived risk is a significant influential factor in health behavior performance [18]. Because hypertension, dyslipidemia, and obesity are known risk factors of CVD, the findings are interpreted as follows: compared to persons without underlying diseases, those with a precursor condition are more sensitive and alert, which affects their anticipated coping behavior. However, diabetes was not a significant factor influencing the anticipated coping behavior in response to MI. Thus, not only persons with a precursor condition to MI but also those with diabetes should be educated on the link to MI, major MI symptoms, and coping behaviors.

Of the socio-demographic variables, age and sex were identified as predictors of coping behavior in response to MI symptoms. Compared to the 20-39 age group, the 40-59 age group was more likely to call-911, while the 60 and older age group was less likely to do so. It is speculated that because the occurrence of MI is higher in the 40-59 age group [25], participants in this age group could obtain a variety of information regarding MI, whereas participants in the 20-39 age group did not consider MI a disease that could afflict them and did not feel the need to seek information, hence the lower level of awareness to call-911. Additionally, it is believed that the likelihood of the coping behavior was lower in the 60 and older age group, as participants in this age group lack an understanding of MI due to aging [6]. Similarly, in a previous study, the rate of calling 911 was the highest in the participants aged between 25 and 64 and the lowest in participants aged 65 and older. In another study, the rate of calling 911 was the highest in the 20-39 age group and the lowest in the 60 and older age group. With respect to sex, females were more likely to call-911 than males. This finding is supported by various studies [11,26]. Accordingly, these findings suggest a need to develop an educational program on appropriate coping behaviors in response to MI symptoms that is specifically designed focusing on males in age groups 20-39 and 60 and older.

This study is of significance in that it laid out a foundation of the application of the HBM by demonstrating that knowledge of the disease (i.e., the structural factor in the HBM) was identified as the most influential factor and that, additionally, sex and age among the socio-demographic variables and hypertension, diabetes, and dyslipidemia among the perceived risks were identified as significant influential factors. Moreover, this article is the first attempt to investigate anticipated coping behavior in response to MI symptoms not in patients but in the general public by using representative survey data. However, because the study was a cross-sectional research, it was not possible to investigate causal relationships, and it cannot be stated that the HBM was completely implemented in the study. Furthermore, caution would be needed in generalizing the study findings because cases with missing data were excluded from the study.

In conclusion, at the onset of MI symptoms, it is absolutely essential to call-911 immediately for early treatment and to avoid delay in the arrival to the hospital. To recognize MI symptoms and cope promptly, it is crucial not only for patients but also for those who care for patients and the general public to have the competence for appropriate coping. For anyone to call-911 immediately at the onset of MI symptoms, the government, healthcare institutions, and educational institutions should actively provide training and campaigns to educate the 20-39 and 60 and older age groups, males, persons with major risk factor of CVD (such as hypertension, dyslipidemia, and obesity), and persons who do not know the various MI symptoms; to be aware of the need to promptly call-911 when witnessing a patient showing MI symptoms; and also to shift the general public’s perception to understand that anyone can call and utilize the 911 service.

## Figures and Tables

**Table 1.** Characteristics of study participants (n=227,740)

Characteristics	Category	n (%)
Sex	Male	102,285 (44.9)
	Female	125,455 (55.1)
Age (yr)	19-39	54,187 (23.8)
	40-59	84,550 (37.1)
	≥60	89,003 (39.1)
Education	≤High school	158,448 (69.6)
	≥College	69,292 (30.4)
Marital status	Married	153,704 (67.5)
	Divorced/widowed/separation	38,639 (17.0)
	Unmarried	35,148 (15.5)
Occupation	Professional/manager/clerk	44,942 (19.8)
	Sales/service/manual	99,244 (43.6)
	Unemployed/housewives	83,291 (36.6)
Household income (10^4^ KRW/mo)	<200	81,252 (36.0)
	200-399	74,575 (33.0)
	≥400	69,861 (31.0)
CVD risk factors	Hypertension	62,203 (27.3)
	Diabetes mellitus	25,097 (11.0)
	Dyslipidemia	39,703 (17.4)
	BMI ≥25 kg/m^2^	59,981 (26.3)
Awareness of MI symptoms	0-1	36,331 (16.0)
	2-3	57,530 (25.3)
	4-5	133,879 (58.8)

KRW, Korean won; CVD, cardiovascular disease; BMI, body mass index; MI, myocardial infarction.

**Table 2.** Characteristics according to coping behavior at MI onset (n=227,740)

Characteristics	Category	Call-911 (n=189,385)	Other (n=38,355)^1^	t or χ^2^	p-value
Sex	Male	84,896 (44.8)	17,389 (45.3)	3.35	0.067
	Female	104,489 (55.2)	20,966 (54.7)		
Age (yr)	19-39	45,356 (23.9)	8,831 (23.0)	146.42	<0.001
	40-59	71,402 (37.7)	13,148 (34.3)		
	≥60	72,627 (38.3)	16,376 (42.7)		
Education	≤High school	131,125 (69.2)	27,323 (71.2)	60.26	<0.001
	≥College	58,260 (30.8)	11,032 (28.8)		
Marital status	Married	128,572 (68.0)	25,132 (65.6)	12.30	<0.001
	Divorced/widowed/separated	31,076 (16.4)	7,563 (19.7)		
	Unmarried	29,520 (15.6)	5,628 (14.7)		
Occupation	Professional/manager/clerk	37,669 (19.9)	7,273 (19.0)	65.54	<0.001
	Sales/service/manual	82,994 (43.9)	16,250 (42.4)		
	Unemployed/housewives	68,502 (36.2)	14,789 (38.6)		
Household income (10^4^ KRW/mo)	<200	66,677 (35.5)	14,575 (38.3)	66.06	<0.001
	200-399	62,559 (33.3)	12,016 (31.6)		
	≥400	58,388 (31.1)	11,473 (30.1)		
CVD risk factors	Hypertension	51,226 (27.0)	10,977 (28.6)	39.64	<0.001
	Diabetes mellitus	20,678 (10.9)	4,419 (11.5)	11.82	0.001
	Dyslipidemia	33,157 (17.5)	6,546 (17.1)	4.31	0.038
	BMI ≥25 kg/m^2^	50,229 (26.5)	9,752 (25.4)	19.77	<0.001
Awareness of MI symptoms	0-1	7,754 (20.2)	28,577 (15.1)	804.74	<0.001
	2-3	10,203 (26.6)	47,327 (25.0)		
	4-5	20,398 (53.2)	113,481 (59.9)		

Values are presented as number (%). MI, myocardial infarction; KRW, Korean won; CVD, cardiovascular disease; BMI, body mass index.

1 Other responses include hospital, oriental hospital, call family, and others.

**Table 3.** Predictors of coping behavior at MI onset

Characteristics	Category	aOR (95% CI)^¹^	p-value
Sex	Male	1.00 (reference)	
	Female	1.02 (1.00, 1.05)	0.033
Age (yr)	19-39	1.00 (reference)	
	40-59	1.07 (1.04, 1.11)	<0.001
	≥60	0.94 (0.90, 0.97)	0.001
CVD risk factors	Hypertension	1.06 (1.03, 1.08)	<0.001
	Diabetes mellitus	1.00 (0.96, 1.04)	0.985
	Dyslipidemia	1.05 (1.02, 1.09)	0.001
	BMI ≥25 kg/m^2^	1.04 (1.02, 1.07)	0.002
Awareness of MI symptoms	0-1	1.00 (reference)	
	2-3	1.23 (1.19, 1.27)	<0.001
	4-5	1.46 (1.41, 1.50)	<0.001

MI, myocardial infarction; aOR, adjusted odds ratio; CI, confidence interval; CVD, cardiovascular disease; BMI, body mass index.

¹ Adjusted for sex, age, education, marital status, occupation, household income, CVD risk factors, awareness of MI symptoms.

## SUPPLEMENTARY MATERIALS

Korean version is available at http://www.e-epih.org/.
