# Supplementary material for: Predictors of anticipated coping behavior at myocardial infarction symptom onset among a nationwide sample of Korean adults
Source: Epidemiol Health. 2021 Jan 3;43:e2021006. doi: 10.4178/epih.e2021006 (PMC8060522; doi:10.4178/epih.e2021006)
Supplement: Supplementary file 1 [file epih-43-e2021006-suppl.docx]

**Predictors of Anticipated Coping Behavior at Myocardial Infarction Symptom Onset among a Nationwide Sample of South Korean Adults**

Kyong Sil Park

School of Nursing, Cheju Halla University, Jeju, South Korea

Correspondence to: Kyong Sil Park, RN, PhD

Address: **38, Halladaehak-ro, Jeju-si, Jeju, Republic of Korea**

Cheju Halla University, School of Nursing

E-mail address: hanibal0505@chu.ac.kr

**ABSTRACT**

**OBJECTIVES:** 건강신념모델을 기반으로 2017년도 지역사회건강조사 자료를 이용하여 심근경색증이 발생한 일반인의 예상 대처 행동을 파악하고, 예상 대처 행동에 미치는 영향 요인을 확인하고자 하였다.

**METHODS:** 수정 요인인 인구사회학적 요인, 건강 지식, 지각된 위험성을 독립변수로, 행동 가능성을 종속변수로 선정하였다. ‘119 신고’를 올바른 예상 대처 행동으로, ‘병원’, ‘한방병원’, ‘가족에게 연락’, ‘기타’는 부적절한 예상 대처 행동으로 구분하여 분석하였다.

**RESULTS:** 총 227,740명의 대상자 중에서 올바른 예상 대처 행동(119 신고)에 대한 비율은 83.2%이었다. 심근경색증 주요 5개 증상 중 비전형적 증상(턱, 목, 등의 통증 및 팔, 어깨의 통증)에 대한 인지를 하더라도 119 신고할 가능성이 낮았다. 반면에 전형적 증상(흉통)에 대한 인지를 한 대상자가 119 신고할 확률을 69.9%로 높았다. 심근경색증 환자 발생 시 일반인의 예상 대처 행동에 미치는 요인은 성별, 연령, 고혈압, 이상지질혈증, 비만, 심근경색증 증상 인지도가 확인되었다.

**CONCLUSIONS:** 심근경색증 환자의 조기 치료 및 내원 시간 감축을 위해 일반인들의 올바른 대처능력이 매우 중요하다. 심근경색증 증상 발현 시 올바른 대처 행동을 위해 20-30대, 60세 이상의 그룹, 남성, 고혈압, 이상지질혈증, 비만 등의 심혈관질환 선행질환이 있는 대상자, 심근경색증 증상을 인지하지 못하는 대상자에게 심근경색증의 전형적, 비전형적 증상에 대해 교육하고, 환자 발생 시 지체없이 응급의료서비스 신고할 수 있으며 누구나 응급의료서비스를 이용할 수 있도록 인식 전환을 위한 홍보활동을 시행해야 한다.

**KEY WORDS:** Myocardial infarction, Heart attack, Cardiovascular disease, Emergency medical service, Behavior, Health belief model

**INTRODUCTION**

국내 심혈관질환의 사망률은 2018년 기준으로 인구 10만 명당 약 62명으로 지속적인 증가 추세를 보이고 있으며, 그 중에서도 심근경색증 및 협심증 등의 허혈성 심장질환의 사망률은 심장질환 사망률의 약 50%를 차지한다[1]. 심근경색증은 다양한 합병증, 장애, 사망을 초래할 수 있으므로 심근경색증 증상 발생 직후 신속한 대처는 환자의 예후를 결정하는 가장 중요한 요인이 된다[2]. 특히, 응급의료서비스(이하 119) 이용은 심근경색증 사망률을 낮추는 독립적인 영향 요인으로 확인되어[3] 심근경색증 환자의 증상 발생 시 신속하고 올바른 119 이용 및 신고는 심근경색증 환자의 사망률 감소 및 예후 개선에 중요하다고 볼 수 있다.

하지만, 심근경색증 증상 발현 후 2시간 이내에 병원 도착한 환자는 약 44%[4], 3시간 이내에 도착한 환자는 약 47.7%로, 50% 이상에서 골든 아워를 초과하는 것으로 나타났다[5]. 자가용 이용 및 가족 연락 등의 부적절한 이송은 병원 도착 지연을 야기시키며[4], 심근경색증에서 가장 중요한 골든 아워를 놓치게 되어 환자의 생존율에도 영향을 미치게 된다[6]. 실제로 심근경색증 환자의 28.8% 만이 119를 이용하였고, 주로 극심한 통증이 있을 경우에만 119를 이용하거나[7], 119를 이용하는데 창피함, 미안함 등[8]의 잘못된 인식을 가지고 있었다.

심근경색증은 환자뿐만 아니라 심근경색증 발생 시 골든 아워 내 적절한 치료를 받을 수 있도록 가족 및 지역사회 일반인의 심근경색증 대처방안에 대한 인지가 중요하다[9]. 최근 일반인을 대상으로 한 연구 결과, 심근경색증의 대표 증상 5가지 중 흉통은 심근경색증의 전형적인 증상으로 인지하고 있으나, 팔, 어깨 등으로 퍼지는 방사통의 증상은 인지율이 낮음을 확인하였다. 또한, 예상되는 대처 행동으로 약 83%가 119신고로 올바른 대처 행동을 선택하였으나[10], 2019년 미국의 119신고 응답률 95.5%와 비교하면[11] 아직 심근경색증의 대처 행동에 대한 인지가 부족함을 확인할 수 있다.

한편, 공공기관에서 일반인을 대상으로 목격자 심폐소생술 교육 및 전국민 홍보가 활발히 이루어지고 있으며, 이 결과로 일반인의 심폐소생술 시행률은 2008년 1.9%에서 2018년도 23.5%로 높은 증가률을 보였다[12]. 그러나, 전문적 지식의 부족과 시행의 두려움 등으로[13] 일반인의 심폐소생술 수행은 여전히 어려움이 존재하고 있다. 또한, 심폐소생술 교육을 받은 후 시간이 경과할수록 심폐소생술 수행 가능성은 낮았다[14]. 이처럼 심폐소생술 수행에 대한 어려움이 존재하므로 일반인의 다양한 특성을 확인하고 그에 적합한 교육 및 홍보가 필요하다.

미국의 경우 일반인을 대상으로 심근경색증 증상 발현 시 적절한 대처 행동에 관련된 연구가 활발하게 진행되어 왔다. 심근경색증 조기증상 인지도와 적절한 대처 행동의 관계를 인구사회학적 요인을 통해 확인하였으며[11], 심근경색증 증상 발현 시 적절한 대처 행동의 예측 요인으로 결혼, 고혈압, 심근경색증의 조기증상 지식 등을 확인하였다[15].

하지만, 국내 심근경색증 관련 연구는 주로 심근경색증 환자를 중심으로 연구가 진행되어 왔다. 심근경색증 환자를 대상으로 심근경색증 증상 인지도, 병원 도착률, 병원 도착 지연 요인[4, 16]을 확인하고, 심뇌혈관질환 및 심폐소생술 교육프로그램이 지식 및 수행 가능성에 영향을 주는[17] 연구가 있었다. 일반인을 대상으로 심근경색 증상 인지도를 확인하고[10], 심뇌혈관질환 및 심폐소생술 교육프로그램이 수행 가능성[16]에 영향을 미치는 연구들이 진행되어 왔다. 특히, 심근경색증 증상 발현 시 대처 행동에 영향을 주는 요인을 파악하는 연구는 심근경색증 환자에 국한하여 연구되어 왔으며, 일반인 대상으로 진행한 연구는 이루어진 바가 없었다.

심근경색증 환자 발생 시 골든 아워 내 적절한 치료를 받기 위해서 심장질환을 가지고 있는 환자뿐만 아니라 지역사회 내 일반인의 올바른 대처 행동이 매우 중요하다[10]. 따라서, 심근경색증 환자 발생 시 최초의 목격자가 빠르고 올바른 대처 행동을 통해 병원전단계의 시간을 줄일 수 있도록 지역사회 내 일반인의 예상 대처 행동을 파악하고, 올바른 대처 행동에 미치는 영향 요인을 파악할 필요가 있다. 따라서, 본 연구는 심근경색증 환자 발생 시 예상되는 대처 행동을 일반인을 대상으로 확인하고자 한다. 인간의 행동의 변화를 설명하는 건강신념모델(Health Belief Model, HBM)을 근거로[18] 구성요소를 대상자의 특성과 연결하여 선정한 후 예상 대처 행동과 관련된 요인을 파악하고자 한다.

**MATERIALS AND METHODS**

**Research design**

이 연구는 심근경색증 환자 발생 시 일반인의 예상 대처 행동을 파악하고, 대처 행동에 미치는 영향 요인을 확인하기 위해 2017년도 지역사회건강조사(Community Health Survey, CHS) 자료를 이용한 단면적 조사연구이다.

**Study** **participants**

연구대상자는 2017년도 지역사회건강조사 원시자료를 활용하였다. 원시자료 228,381명의 대상자 중 심근경색증 증상 문항의 결측자 100명과 심근경색증 대처 방법 문항의 결측자 541명을 제외한 최종 227,740명을 연구대상자로 선정하였다.

**Ethics statement**

연구자료는 지역사회건강조사 웹 사이트를 통해 신청을 하여 승인받은 후 지역사회건강조사로부터 대상자의 개인 정보가 삭제된 원시자료를 받았다.

**Measurements**

건강신념모델은 흡연, 운동, 의료 서비스 이용과 같은 건강관련 행위를 설명하는 중요 예측인자로 활용되고 있으며, 최근 다양한 분야에서 행위 예측에 적용되고 있다[18]. 본 연구는 지역사회건강조사 수집자료를 활용한 선행연구에 근거하여[14] 건강신념모델의 모든 구성요소를 확인하기에 제한이 있어 수정 요인인 인구사회학적 요인, 건강지식, 지각된 위험성을 독립변수로, 예상 대처 행동을 종속변수로 선정하였다.

인구사회학적 요인에 성별, 연령, 교육수준, 결혼상태, 직업, 수입, 구조적 요인에 심근경색 조기 증상 인지도, 지각된 위험성에 고혈압, 당뇨, 이상지질혈증, 비만을 선정하였다. 직업은 전문행정관리 및 사무직(관리자, 전문가 및 관련종사자, 사무종사자), 판매서비스직 및 기능단순노무직(서비스종사자, 판매종사자, 농림어업종사자, 기능원 및 관련기능종사자, 장치, 기계조작 및 조립종사자, 단순노무종사자, 군인), 무직 및 주부(무직, 주부, 학생)로 범주화하였다. 심혈관질환 관련 선행질환 중 고혈압, 당뇨, 이상지질혈증은 ‘의사의 진단 여부’ 또는 ‘현재 치료 여부’에 ‘예’로 응답한 경우 질환이 있는 것으로 구분하였다. 지역사회건강조사 가이드라인에 따라 고혈압은 수축기혈압 ≥ 140mmHg 또는 이완기 혈압 ≥ 90mmHg, 당뇨는 공복혈당 ≥ 126mg/dL 또는 경구포도당부하검사 2시간 후 혈당 ≥ 200mg/dL 또는 당화혈색소 ≥ 6.5%, 이상지질혈증은 저밀도지단백(low density lipoprotein, LDL)-콜레스테롤 ≥ 160mg/dL 또는 고밀도지단백(high density lipoprotein, HDL)-콜레스테롤 < 40mg/dL 또는 총콜레스테롤 ≥ 240mg/dL 또는 중성지방 ≥ 200mg/dL 으로 정의된다[19]. 비만은 체질량지수(Body Mass Index, BMI) ≥ 25 kg/㎡로 정의하였다[20-21].

건강신념모델의 구조적 요인은 질병에 대한 지식에 해당한다[18]. 본 연구에서 질병에 대한 지식은 심근경색증의 조기 증상의 지식을 선정하였고, “심근경색증의 증상이라고 생각하는 문항은?” : 1) 턱, 목, 등에 통증이나 답답함, 2) 힘이 없으며, 어지럽고, 울렁거리거나 식은땀, 3) 가슴에 통증이나 압박감 또는 짓누르는 느낌, 4) 갑자기 팔, 어깨에 통증이나 불편감, 5) 갑자기 숨이 참으로 구성되어 있으며, ‘예’ 또는 ‘아니오’로 응답하도록 하였다. 심근경색증 증상 문항에서 ‘모름’으로 응답한 경우는 심근경색증에 대한 지식을 모른다고 판단하여 ‘아니오’로 재코딩하였다. ‘응답거부’로 응답한 경우는 결측 처리하여 대상자 선정에서 제외하였다.

종속변수는 심근경색증 환자 발생 시 예상 대처 행동을 선정하였다. ‘누군가 심근경색증 증상을 보인다면, 가장 먼저 무엇을 해야 한다고 생각합니까?’의 문항에서 질병관리본부 가이드라인에 따라[22] ‘119 신고’를 올바른 대처 행동으로, ‘병원’, ‘한방병원’, ‘가족에게 연락’, ‘기타’는 부적절한 대처 행동으로 구분하였다.

**Data analysis**

자료분석은 SPSS program version 20을 이용하였다. 명목변수는 사례수와 백분율로, 연속변수는 평균 및 표준 편차로 제시하였다. 심근경색증 환자 발생 시 예상 대처 행동을 확인하기 위해 카이제곱 검정을 실시하였고, 예상 대처 행동에 영향을 미치는 요인을 확인하기 위해서 다중 로지스틱 회귀분석(Multivariate logistic regression)을 실시하였다. 통계적 유의성은 *p* <.05으로 정하였다.

**RESULTS**

인구사회학적 특성을 살펴보면 여성은 55.1%로 남성보다 많았고, 연령은 60대 이상이 39.1%로 높았다. 학력에서 고졸자 이하가 69.6%로 높았고, 결혼상태에서 기혼자가 67.5%였다. 직업은 판매서비스직 및 기능단순노무직이 43.6%이며, 월 소득은 200만원 미만이 36.0%로 높았다. 심혈관질환의 주요 위험요인이 되는 고혈압은 27.3%, 당뇨 11.0%, 이상지질혈증 17.4%, BMI 25 ㎏/㎡이상인 대상자 26.3%였다. 심근경색증 주요 증상 5가지 중 4-5개 인지한 대상자는 58.8%였다(Table 1).

심근경색증 증상 발현 시 예상되는 대처 행동에 대해 119 신고 그룹과 그 외 그룹의 특성을 비교한 결과, 연령이 40대 이상인 경우, 고등학교 졸업 이하인 경우, 기혼자인 경우, 판매서비스직 및 기능단순노무직인 경우, 월 소득이 200만원 미만인 경우, 고혈압, 당뇨, 이상지질혈증, 비만이 있는 경우, 심근경색증 조기증상을 인지한 경우 119에 전화하는 비율이 높았다(Table 2).

심근경색증 주요 증상 5가지와 예상 대처 행동을 비교한 결과, ‘턱, 목, 등에 통증이나 답답함’을 인지하고 있는 대상자가 119에 신고할 비율은 53.6%, ‘힘이 없으며, 어지럽고, 울렁거리거나 식은땀’을 인지하고 있는 대상자가 119에 신고할 비율은 58.6%, ‘가슴에 통증이나 압박감 또는 짓누르는 느낌’을 인지하고 있는 대상자가 119에 신고할 비율은 69.9%, ‘갑자기 팔, 어깨에 통증이나 불편감’을 인지하고 있는 대상자가 119에 신고할 비율은 45.7%, ‘갑자기 숨이 참’을 인지하고 있는 대상자가 119에 신고할 비율은 65.7%였다(Figure 1).

심근경색증 증상 발현 시 대처 행동에 영향을 미치는 요인을 확인한 결과, 남성보다 여성(odds ratio[OR] 1.02, 95% confidence interval [CI] 1.00-1.05)이 높았고, 20-30대보다 40-50대(OR 1.07, 95% CI 1.04-1.11)가 높았으며, 60대 이상(OR 0.94, 95% CI 0.90-0.97)이 낮았다. 고혈압(OR 1.06, 95% CI 1.03-1.08), 이상지질혈증(OR 1.05, 95% CI 1.02-1.09), BMI 25㎏/㎡ 이상(OR 1.04, 95% CI 1.02-1.07)인 대상자가 정상에 비해 높았다. 심근경색증 주요 증상 5가지 중 1개 이하로 인지한 대상자에 비해 2-3개 인지한 대상자가(OR 1.23, 95% CI 1.19-1.27) 유의하게 높았고, 4-5개 인지한 사람이(OR 1.46, 95% CI 1.41-1.50) 높았다(Table 3).

**DISCUSSION**

한국에서 심근경색증 환자 발생 시 예상되는 일반인의 대처 행동으로 올바른 대처 행동인 119 신고가 83.2%로 나타났으며, 2010년 결과 67%[23]보다 상승된 수치를 보였다. 이러한 결과는 전국민 교육 및 홍보의 결과로 볼 수 있으나, 미국의 119 신고율 95.5%보다[11] 상대적으로 낮으며, 약 16.8%는 병원 및 한방병원 방문, 가족에게 연락 등의 부적절한 대처 행동을 함으로써 심근경색증 환자의 병원내 도착 지연을 발생시킬 수 있다. 119 구급차가 단순하게 운송 수단이 아니며, 진단 및 치료를 제공하는 중요한 1차 의료서비스임을[7] 강조하는 교육 및 홍보가 필요하다.

심근경색증 증상 발현 시 일반인의 예상 대처 행동에 미치는 요인을 확인한 결과, 심근경색증 조기 증상 인지도가 대처 행동을 예측하는 가장 큰 관련 요인이었다. 홍콩의 경우 부적절한 대처 행동에 심근경색증의 조기 증상 인지가 영향 요인으로 밝혀져[15] 본 연구결과와 같았다. 더불어, 심근경색증 주요 증상을 알고 있으나 올바른 대처 행동을 하는 경우를 확인해보면, 턱, 목, 등의 통증을 알고 있는 경우 119 신고가 53.6%, 팔과 어깨의 통증 증상을 알고 있는 경우 119 신고가 45.7% 밖에 되지 않았다. 반면에 흉통을 심근경색증 증상으로 인지하고 있는 겨우 119신고는 69.9%로 가장 높았다. 즉, 심근경색증의 증상을 잘 알고 있더라도 부적절한 대처를 할 가능성이 높으며, 전형적 증상보다 비전형적 증상의 경우 더 부적절한 대처를 할 가능성이 높다고 해석할 수 있다. 유사하게 선행연구에서 흉통과 목통증의 증상의 인지가 부적절한 행동에 예측 요인으로 확인되었다[15]. 실제 심근경색증 환자의 질적 연구 결과, 비전형적 증상에 대한 이해가 부족하며, 응급의료서비스를 이용하는 것에 창피함, 미안함 등의 감정을 느끼는 등의 잘못된 인식을 가지고 있었다[8]. 또한, 119 구급차를 극심한 통증이 있을 때만 이용하는 것으로 잘못 판단하고 있었다[7]. 따라서, 올바른 대처 행동인 119 신고를 정착시키기 위해서 심근경색증의 전형적 증상뿐만 아니라 비전형적 증상도 함께 중요하게 교육시켜야 하며, 위급상황에서 누구나 119 신고 또는 이용할 수 있는 인식 제고를 위한 교육 및 홍보 활동을 적극적으로 시행해야 하겠다.

지각된 위험성에 해당하는 고혈압, 이상지질혈증, 비만인 대상자는 심근경색증 증상 발현 시 대처 행동을 예측하는 요인으로 밝혀졌다. 선행연구에서 고혈압이 부적절한 치료 추구 행동에 유의한 영향 요인으로 확인되었으며[15], 심근경색증 또는 협심증이 있는 대상자는 심폐소생술 수행 가능성이 높았다[14]. 직접 비교는 어려우나, 심뇌혈관질환 인지도에 영향을 미치는 요인으로 고혈압, 이상지질혈증, 뇌졸중, 심근경색증, 협심증이 있었다[24]. 건강신념모델에 따르면 지각된 위험성은 건강 행동 실천에 유의한 영향요인으로 볼 수 있다[18]. 고혈압, 이상지질혈증, 비만은 심혈관질환의 위험 요인으로 알려져 있기 때문에 선행질환을 가지고 있는 대상자는 정상인 대상자들에 비해 높은 민감성과 심각성을 가지고 있어 대처 행동에도 영향을 미치는 것으로 해석된다. 하지만, 당뇨는 심근경색증 대처 행동에 유의한 영향요인이 아니었다. 따라서, 심근경색증 선행 질환을 가지고 있는 대상자뿐만 아니라 당뇨를 가지고 있는 대상자들에게 심근경색증과의 연관성 교육 및 심근경색증 주요 증상, 대처 행동에 대해 교육할 필요가 있겠다.

인구사회학적 변수인 연령과 성별은 심근경색증 증상 발현 시 대처 행동을 예측하는 요인으로 확인되었다. 연령에서 40-50대는 20-30대에 비해 119에 연락할 가능성이 높았으나, 60대 이상은 20-30대에 비해 119에 연락할 가능성이 낮았다. 40-50대는 심근경색증 발생률이 높으므로[25] 질환에 대한 다양한 정보 습득이 가능했으나, 20-30대는 자신과 거리가 먼 질환으로 생각하고 필요성을 못 느꼈기 때문에 119 신고에 대한 인식이 부족할 것으로 판단된다. 또한, 60세 이상의 연령군은 노화로 인하여 심근경색증에 대한 이해 부족[6]으로 대처 행동에 대한 가능성도 낮은 것으로 해석된다. 선행연구에서도 유사하게 25-64세의 119 신고율이 가장 높았고, 65세 이상에서 가장 낮았다. 또 다른 연구에는 20-30대의 119 신고율이 가장 높고, 60세 이상에서 가장 낮았다. 성별에서 여성은 남성에 비해 119에 연락할 가능성이 높았으며, 다양한 선행연구 결과에서 이를 뒷받침해주고 있다[11, 26]. 따라서, 20-30대, 60세 이상의 남성을 중심으로 심근경색증의 올바른 대처 행동에 대한 맞춤형 프로그램이 필요함을 시사하고 있다.

본 연구는 건강신념모델을 근거로 구조적 요인인 질병에 대한 지식이 가장 큰 영향 요인으로 확인되었고, 인구사회학적 요인에서 성별, 연령, 지각된 위험성에 고혈압, 당뇨, 이상지질혈증이 유의한 영향요인임을 확인함으로써 모델 적용의 근거를 마련하였다는 의의가 있다. 또한, 대표성을 가지고 있는 자료를 활용하여 심근경색증 대상자가 아닌 일반인들의 예상 대처 행동을 확인하는 처음 시도된 논문이다. 하지만, 단면적 조사연구이므로 인과관계를 확인할 수 없기 때문에 건강신념모델을 완전히 구현하였다고 할 수 없고, 결측 자료를 제외하였기 때문에 연구결과를 일반화하는데 신중함이 있어야 하겠다.

심근경색증 증상이 나타났을 때 조기 치료 및 내원 시간을 감축시키기 위해서 119에 빠른 신고가 절실히 필요하다. 심근경색증의 증상을 인지하고 빠른 대처를 위해 환자뿐만 아니라 환자를 돌보고 목격하는 일반인들의 올바른 대처능력이 매우 중요하다. 심근경색증 증상 발현 시 119 신고를 위해 20-30대, 60세 이상의 그룹, 남성, 고혈압, 이상지질혈증, 비만 등의 심혈관질환 선행질환이 있는 대상자, 심근경색증 증상을 인지하지 못하는 대상자에게 심근경색증의 다양한 증상들을 교육하고, 심근경색증 환자 발견 시 지체없이 119 신고를 할 수 있도록 교육하며, 누구나 119 신고 또는 이용할 수 있는 인식의 전환을 위해 정부, 의료기관, 교육기관 등이 적극적으로 교육 및 홍보활동을 시행해야 하겠다.

**REFERENCES**

1. Statistical Korea. The cause of Death Statistics; 2018 [cited 2020 Oct 20] Available from <http://kostat.go.kr/portal/korea/kor_nw/1/6/1/index.board?bmode=read&bSeq=&aSeq=377606&pageNo=1&rowNum=10&navCount=10&currPg=&searchInfo=&sTarget=title&sTxt=>
2. Oh HS, Jang KS, Jeong MH. Decision tree model of treatment-seeking delay among patients with acute coronary syndrome. Korean Journal of Health Promotion and Disease Prevention 2008;8:235-244.
3. Kim SJ, Kim CJ, Lee ES, Kim YJ, Kim MC, Kim JH, et al. Comparison of major cardiac and cerebrovascular events in patients with acute myocardial infarction according to the use of emergency medical service during one-year clinical follow-up. Journal of the Korean Society of Emergency Medicine 2020;31:181-190.
4. Ahn HM, Kim H, Lee KS, Lee JH, Jeong HS, Chang SH, et al. Hospital Arrival Rate within Golden Time and Factors Influencing Prehospital Delays among Patients with Acute Myocardial Infarction. Journal of Korean Academy of Nursing 2016;46:804-812.
5. Ministry of Health & Welfare. Regional Cardiocerebrovascular Center Operating Instructions; 2019 [cited 2020 Oct 20] Available from <http://www.mohw.go.kr/react/jb/sjb030301vw.jsp?PAR_MENU_ID=03&MENU_ID=032903&CONT_SEQ=349417&page=1>
6. Won MS, Shin NM, Kim E. Gender Differences in Delay Seeking Treatment and Related Experiences in Patients with Acute Myocardial Infarction. Korean Journal of Adult Nursing 2016;28:459-469.
7. Woo SH, Yun KH, Lee MR, Kim EK, Ko JS, Lee SJ, et al. Emergency Medical Service Use among Patients with Acute ST-segment Elevation Myocardial Infarction in Jeonbuk Province. Korean Journal of Medicine 2016;90:507-513.
8. Hwang SY, Kweon YR, Kim AL. Cardiovascular Risk Factors and Decision to Seek Treatment among Middle-aged Men with Acute Myocardial Infarction. Journal of Korean Academic Society of Adult Nursing 2010;22:537-551.
9. Ministry of Health & Welfare. Cardiovascular Disease Management Comprehensive Plan; 2018 [cited 2020 Oct 20] Available from <http://www.mohw.go.kr/react/al/sal0301vw.jsp?PAR_MENU_ID=04&MENU_ID=0403&CONT_SEQ=345946&page=1>
10. Park KS. Factors affecting awareness of myocardial infarction symptoms among the general public. Epidemiology and Health 2020;42:e2020032.
11. Mahajan S, Valero-Elizondo J, Khera R, Desai NR, Blankstein R, Blaha MJ, et al. Variation and Disparities in Awareness of Myocardial Infarction Symptoms Among Adults in the United States. JAMA Network Open 2019;2:e1917885.
12. Yoon HJ, Park SJ, An JY, Lee YK. Key Findings of the Sudden Cardiac Arrest (SCA) Survey from 2006 to 2018. Public Health Wkly Rep 2020;13:77-90 (Korean).
13. Park SJ, Lee MJ, Park YS. Difference of Awareness and Barrier about Bystander Cardiopulmonary Resuscitation between Adult and Geriatric Population. Journal of the Korean Society of Emergency Medicine 2017;28:620-627.
14. Kang MS, Lee EY, Choi BY. Factors influencing the possibility to perform cardiopulmonary resuscitation among lay cardiopulmonary resuscitation trainees: Using the data of the 2016 Community Health Survey. Korean J Health Educ Promot 2020;37:85-98.
15. Chau PH, Moe G, Lee SY, Woo J, Leung AY, Chow CM, et al. Low level of knowledge of heart attack symptoms and inappropriate anticipated treatment-seeking behaviour among older Chinese: a cross-sectional survey. J Epidemiol Community Health 2018;72:645-652.
16. Han EJ, Kim JS. Effects of Symptom Recognition and Health Behavior Compliance on Hospital Arrival Time in Patients with Acute Myocardial Infarction. Journal of Korean Academic Society of Adult Nursing 2015;27:83-93.
17. Song JK, Park HK, Hong SC. Effects of an Educational Program for the High Risk Group of Cardio-cerebrovascular Disease: Awareness of the Warning Signs and Symptoms of Acute Myocardial Infarction and Stroke in the Aged at Senior Centers. J Agric Med Community Health 2015;40:126-136.
18. Shojaei S, Farhadloo R, Aein A, Vahedian M. Effects of the health belief model (HBM)-based educational program on the nutritional knowledge and behaviors of CABG patients. The Journal of Tehran University Heart Center 2016;11:181-186.
19. Korea Centers for Disease Control and Prevention. Community Health Survey 2017; 2020 [cited 2020 Oct 20] Available from <https://www.daedeok.go.kr/board/binary/CHC_000005/2030138.pdf>
20. Khan SS, Ning H, Wilkins JT, Allen N, Carnethon M, Berry JD, et al. Association of body mass index with lifetime risk of cardiovascular disease and compression of morbidity. JAMA cardiology 2018;3:280-287.
21. Korean Society for the Study of Obesity. Obesity fact sheet 2019 [cited 2020 Mar 31]. Available from: https://www.kosso.or.kr/file/2019_Obesity_Fact_Sheet_web_kor.pdf (Korean).
22. Korea Centers for Disease Control and Prevention. Sudden Cardiac Death Survey 2006-2017; 2020 [cited 2020 Oct 20] Available from <http://www.cdc.go.kr/board.es?mid=a20503050000&bid=0021&act=view&list_no=142010>
23. Kim HS, Lee H, Kim K, Park HK, Park KS, Kang GW, et al. The general public’s awareness of early symptoms of and emergency responses to acute myocardial infarction and related factors in south korea: a national public telephone survey. Journal of epidemiology 2016;26:233-241.
24. Lee YH, Noh SE. Factors Related to Awareness of Cardio-cerebrovascular Disease among Korean Adults: the 2013 Community Health Survey. Korean J Health Promot 2017;17:99-108.
25. Holly Felix MRN, Rowland B, Long CR, Bursac Z, McElfish PA. Level of Recommended Heart Attack Knowledge among Native Hawaiian and Pacific Islander Adults in the United States. Hawai'i Journal of Medicine & Public Health 2019;78:61.
26. Fang J, Gillespie C, Keenan NL, Greenlund KJ. Awareness of heart attack symptoms among US adults in 2007, and changes in awareness from 2001 to 2007. Future cardiology 2011;7:311-320.

Table 1. Characteristics of study participants (n=227,740)

| Characteristics | Category | N (%) |
| --- | --- | --- |
| Sex | Men | 102,285 (44.9) |
|  | Women | 125,455 (55.1) |
| Age (years) | 19-39 | 54,187 (23.8) |
|  | 40-59 | 84,550 (37.1) |
|  | ≥60 | 89,003 (39.1) |
| Education | High school | 158,448 (69.6) |
|  | ≥College | 69,292 (30.4) |
| Marital status | Married | 153,704 (67.5) |
|  | Divorced/widowed/separation | 38,639 (17.0) |
|  | Unmarried | 35,148 (15.5) |
| Occupation | Professional/manager/clerk | 44,942 (19.8) |
|  | Sales/service/manual | 99,244 (43.6) |
|  | Unemployed/housewives | 83,291 (36.6) |
| Household income  (×10K KRW/month) | <200 | 81,252 (36.0) |
|  | 200-399 | 74,575 (33.0) |
|  | ≥400 | 69,861 (31.0) |
| CVD risk factors | Hypertension | 62,203 (27.3) |
|  | Diabetes mellitus | 25,097 (11.0) |
|  | Dyslipidemia | 39,703 (17.4) |
|  | BMI ≥25 ㎏/㎡ | 59,981 (26.3) |
| Awareness of MI symptoms | 0-1 | 36,331 (16.0) |
|  | 2-3 | 57,530 (25.3) |
|  | 4-5 | 133,879 (58.8) |

Values are presented as number (%).

CVD, cardiovascular disease; BMI, body mass index; MI, myocardial infarction

1Other responses include hospital, oriental hospital, call family, and others.

Table 2. Characteristics according to coping behavior at myocardial infarction onset (n=227,740)

| Characteristics | Category | Call-911  (n=189385, 83.2%) | Other¹  (n=38355, 16.8%) | t or ｘ² | *p* |
| --- | --- | --- | --- | --- | --- |
|  |  | N (%) or M±SD | N (%) or M±SD |  |  |
| Sex | Men | 84,896 (44.8) | 17,389 (45.3) | 3.35 | 0.067 |
|  | Women | 104,489 (55.2) | 20,966 (54.7) |  |  |
| Age (years) | 19-39 | 45,356 (23.9) | 8,831 (23.0) | 146.42 | <.001 |
|  | 40-59 | 71,402 (37.7) | 13,148 (34.3) |  |  |
|  | ≥60 | 72,627 (38.3) | 16,376 (42.7) |  |  |
| Education | High school | 131,125 (69.2) | 27,323 (71.2) | 60.26 | <.001 |
|  | ≥College | 58,260 (30.8) | 11,032 (28.8) |  |  |
| Marital status | Married | 128,572 (68.0) | 25,132 (65.6) | 12.30 | <.001 |
|  | Divorced/widowed/separation | 31,076 (16.4) | 7,563 (19.7) |  |  |
|  | Unmarried | 29,520 (15.6) | 5,628 (14.7) |  |  |
| Occupation | Professional/manager/clerk | 37,669 (19.9) | 7,273 (19.0) | 65.54 | <.001 |
|  | Sales/service/manual | 82,994 (43.9) | 16,250 (42.4) |  |  |
|  | Unemployed/housewives | 68,502 (36.2) | 14,789 (38.6) |  |  |
| Household income (×10K KRW/month) | <200 | 66,677 (35.5) | 14,575 (38.3) | 66.06 | <.001 |
|  | 200-399 | 62,559 (33.3) | 12,016 (31.6) |  |  |
|  | ≥400 | 58,388 (31.1) | 11,473 (30.1) |  |  |
| CVD risk factors | Hypertension | 51,226 (27.0) | 10,977 (28.6) | 39.64 | <.001 |
|  | Diabetes mellitus | 20,678 (10.9) | 4,419 (11.5) | 11.82 | 0.001 |
|  | Dyslipidemia | 33,157 (17.5) | 6,546 (17.1) | 4.31 | 0.038 |
|  | BMI ≥25 ㎏/㎡ | 50,229 (26.5) | 9,752 (25.4) | 19.77 | <.001 |
| Awareness of MI symptoms | 0-1 | 7,754 (20.2) | 28,577 (15.1) | 804.74 | <.001 |
|  | 2-3 | 10,203 (26.6) | 47,327 (25.0) |  |  |
|  | 4-5 | 20,398 (53.2) | 113,481 (59.9) |  |  |

Values are presented as number (%).

CVD, cardiovascular disease; BMI, body mass index; MI, myocardial infarction

¹Other responses include hospital, oriental hospital, call family, and other.

**Figure 1.** Comparison of correct anticipated coping behavior (call-911) with each symptom of myocardial infarction.

Table 3. Predictors of coping behavior at myocardial infarction onset

| Characteristics | Category | aOR (95% CI)¹ | *p* |
| --- | --- | --- | --- |
| Sex | Men | ref |  |
|  | Women | 1.02 (1.00-1.05) | 0.033 |
| Age (years) | 19-39 | ref |  |
|  | 40-59 | 1.07 (1.04-1.11) | <.001 |
|  | ≥60 | 0.94 (0.90-0.97) | 0.001 |
| CVD risk factors | Hypertension | 1.06 (1.03-1.08) | <.001 |
|  | Diabetes mellitus | 1.00 (0.96-1.04) | 0.985 |
|  | Dyslipidemia | 1.05 (1.02-1.09) | 0.001 |
|  | BMI ≥25 ㎏/㎡ | 1.04 (1.02-1.07) | 0.002 |
| Awareness of MI symptoms | 0-1 | ref |  |
|  | 2-3 | 1.23 (1.19-1.27) | <.001 |
|  | 4-5 | 1.46 (1.41-1.50) | <.001 |

CVD, cardiovascular disease; BMI, body mass index; MI, myocardial infarction

¹Adjusted for sex, age, education, marital status, occupation, household income, CVD risk factors, awareness of MI symptoms
